# Ensemble ecological niche modeling of West Nile virus probability in Florida

**DOI:** 10.1371/journal.pone.0256868

**Published:** 2021-10-08

**Authors:** Sean P. Beeman, Andrea M. Morrison, Thomas R. Unnasch, Robert S. Unnasch

**Affiliations:** 1 Center for Global Health Infectious Disease Research, University of South Florida, Tampa, Florida, United States of America; 2 Bureau of Epidemiology, Division of Disease Control and Health Protection, Florida Department of Health, Tallahassee, Florida, United States of America; Instituto Federal de Educacao Ciencia e Tecnologia Goiano - Campus Urutai, BRAZIL

## Abstract

Ecological Niche Modeling is a process by which spatiotemporal, climatic, and environmental data are analyzed to predict the distribution of an organism. Using this process, an ensemble ecological niche model for West Nile virus habitat prediction in the state of Florida was developed. This model was created through the weighted averaging of three separate machine learning models—boosted regression tree, random forest, and maximum entropy—developed for this study using sentinel chicken surveillance and remote sensing data. Variable importance differed among the models. The highest variable permutation value included mean dewpoint temperature for the boosted regression tree model, mean temperature for the random forest model, and wetlands focal statistics for the maximum entropy mode. Model validation resulted in area under the receiver curve predictive values ranging from good [0.8728 (95% CI 0.8422–0.8986)] for the maximum entropy model to excellent [0.9996 (95% CI 0.9988–1.0000)] for random forest model, with the ensemble model predictive value also in the excellent range [0.9939 (95% CI 0.9800–0.9979]. This model should allow mosquito control districts to optimize West Nile virus surveillance, improving detection and allowing for a faster, targeted response to reduce West Nile virus transmission potential.

## Introduction

Ecological Niche Modeling (ENM), also known as Environmental Niche Modeling, Species Distribution Modeling, or Habitat Suitability Modeling involves the use of computer algorithms to analyze features of a set of geographic locations that together represent a known niche of an organism of interest, with the goal of predicting its distribution across a defined geographic region. These algorithms make use of presence, presence and absence, or presence and pseudoabsence (PA) data of the organism of interest, along with spatial and temporal climatic and environmental data in the known niche to develop a model that describes a niche favorable for supporting the organism in question. This model is then compared to other geospatial regions or even future climate models to predict their suitability as a potential habitat for the organism.

The concept of ecological drivers of species distribution which underlies ENMs can be traced back to the 1800s [1, 2]. This concept continued to mature through the 1900s with the work of Andreas Schimper [3], Frederic Clements [4, 5], and Robert Whitaker [6–8] with modern ENMs having a common ancestor—the 1981 study by Elgene O. Box that predicted vegetation changes based upon climate variables [9]. This publication presented one of the first computer based ENMs.

Since 2000, a combination of factors, including an increase in computing power, development and refinement of Geographic Information Systems, improvement in resolution, accuracy, availability of remote sensing data and the advent of machine learning have revolutionized ENM. The ability to access and download accurate and detailed georeferenced remote sensing data representing climate and environmental variables for a region opened the door to new biogeographical analyses. Originally used for determining potential ecological niches of plants and animals, ENMs are now being used in a variety of applications, creating new specializations across many fields. One such specialization is disease biogeography, which examines and predicts the spatial and temporal distribution of disease by employing the skills and tools of epidemiologists and ecologists. ENMs are now being utilized within this specialty to determine risk of disease for a given population or habitat for disease within a given geographic range.

Vector-borne disease is a significant cause of morbidity and mortality in the world today, comprising 17% of reported infectious disease worldwide with over 700,000 deaths annually [10]. Mosquitoes are responsible for transmitting the majority of vector-borne pathogens, hosting both viral and parasitic agents of disease [10]. While dengue and malaria are responsible for the greatest morbidity and mortality worldwide [11, 12], West Nile Virus (WNV) is the mosquito-borne disease with the greatest geographic distribution worldwide [13].

The State of Florida is unique in the United States in that, due to its climate and environment, year-round transmission of many mosquito-borne viruses is observed [14–17]. Three mosquito-borne viruses commonly found within Florida are WNV, Eastern Equine Encephalitis Virus (EEEV), and St. Louis Encephalitis Virus (SLEV) [18]. In addition, travel related and sporadic autochthonous cases of chikungunya, dengue, malaria, and Zika virus are not unusual, due to Florida’s proximity to the Caribbean, extensive cruise ship traffic, and air travel through international airports [19–22].

To protect human and animal populations from these diseases, mosquito control programs (MCP) have been developed throughout Florida. Surveillance programs employ sentinel chickens, light traps, gravid traps, resting traps, BG Sentinel traps, and larval dipping. Within Florida, 63 state-approved MCPs exist, with programs managed at the county, city, or special taxing district level. However, the types of surveillance techniques, sampling design, frequency of sampling, and availability of resources, personnel, and funding vary drastically among MCPs. Implemented in 1978, the first sentinel chicken surveillance sites were selected based on proximity to documented human cases of SLEV that occurred during outbreaks between 1959 and 1977 [23], with later sites selected by MCPs based upon general recommendations developed by the Florida Interagency Arbovirus Task Force [24], or for their maintenance and sampling convenience. As WNV has generally supplanted SLEV in Florida [25, 26], developing models that identify habitats most likely to harbor WNV would allow surveillance activities to focus on such high-probability areas, increasing the effectiveness of surveillance for WNV in Florida.

Here, we report the development of an ensemble ENM based upon integration of three independent machine learning models—Boosted Regression Tree (BRT), Random Forest (RF), and Maximum Entropy (Maxent)—to identify areas most appropriate for WNV surveillance activity in Florida. This information can be used by MCPs to optimize placement of sentinel chicken coops within their area of operation, increasing their ability to detect WNV activity while reducing operating costs by eliminating unnecessary, misplaced, or redundant locations.

## Methods

### Study location

The state of Florida is a peninsular land form in the Southeastern United States between 24.5- and 31-degrees north latitude and 80- and 87.5-degrees west longitude. Florida is bordered by the state of Georgia to the north, the state of Alabama to the northwest, the Atlantic Ocean to the east, the Gulf of Mexico to the west, and the Straits of Florida connects the two bodies of water to the south. The climate ranges from subtropical in the north and central regions to tropical in the south [27]. Florida has an area of 170,300 km^2^, making it the 22^nd^ largest state in the United States geographically [28], while being the 3^rd^ most populous state, with over 21 million people [29]. Florida’s elevation ranges from sea level to 345 feet above sea level [30] with approximately 18% (30,424 km^2^) of the state covered by water [31]. Bodies of water were excluded *a priori* as unacceptable locations for coop placement.

### Software

Initial raster data analysis, conversion of spatial data for use in R, and creation of raster maps for publication was conducted in ArcGIS Pro version 2.6.3 [32] utilizing SDM toolbox Pro version 0.9.1 [33]. Ecological niche modeling was conducted using R statistical computing software version 4.0.2 [34] with RStudio version 1.3.1056 [35] utilizing the packages SDMtune 1.1.0 [36], dismo version 1.1–4 [37], raster version 3.1–5 [38], pROC version 1.16.2 [39], zealot version 0.1.0 [40], rJava version 0.9–13 [41], and readr 1.3.1 [42]. Presence data was compiled using Microsoft Excel 2019.

### Data

The Florida Department of Health provided data for the thirty-one sentinel chicken surveillance programs that were operational during the 2014–2018 timeframe. Records provided included collection date, site name, laboratory sample number, latitude, and longitude for each coop. Latitude and longitude of the sentinel chicken coops are recorded by the operating MCP using Global Positioning System (GPS) equipment and provided to the Florida Department of Health for tracking. The number of chickens at each location varied by MCP and ranged from 3 to 10 chickens per coop. Not all MCPs conduct sentinel chicken surveillance year-round, so positive chicken locations for the study were selected for the period in which all programs were operating—Julian weeks 18 to 49. Samples are collected weekly by the MCP and sent to the Florida Department of Health Bureau of Public Health Laboratories—Tampa for WNV, EEEV, and SLEV testing with results provided to the MCP. Chickens testing seropositive for any of the three viruses are removed from the coop and replaced as needed. During the five-year period of this study, 2102 sentinel chickens tested seropositive for WNV at 269 locations. This data was compiled in Microsoft Excel and converted into a comma separated values (CSV) file format for import into ArcGIS Pro and R.

Topologically Integrated Geographic Encoding and Referencing (TIGER) United States state, county, and road shapefiles were used to develop Florida state and county borders [43, 44] along with Florida primary and secondary roads [45]. Primary roads are predominantly interstate highways while secondary roads are comprised of U.S., State, and County Highways.

Land cover characteristics were provided by the 2016 National Land Cover Database (NLCD) [46]. The NLCD is a 30-meter resolution raster representing land cover characteristics of the continental United States. Land cover characteristics are divided into 16 classes based on a modified Anderson Level II classification system [47]. Focal summary statistics rasters for both forest and wetland land cover were developed from reclassified binary presence/absence rasters derived from the NLCD.

Parameter-elevation Relationships on Independent Slopes Model (PRISM) historical climate data provided 30-year (1981–2010) normals for precipitation, mean temperature, and mean dewpoint temperature at 800-meter resolution [48]. Precipitation data was provided in millimeters with mean temperature and mean dewpoint temperature provided in degrees Celsius.

Historical remote sensing phenology imagery from Collection 6 of the Moderate Resolution Imaging Spectroradiometer (MODIS) located aboard the National Space and Aeronautics Administration Aqua satellite provided annual values of the maximum and amplitude of the normalized difference vegetation index (NDVI) for the study region at a 250-meter resolution [49, 50]. This data was used to develop rasters representing 15-year (2004–2018) means of the maximum and amplitude NDVI values.

The digital elevation model (DEM) for the state of Florida is a mosaic DEM developed by the University of Florida GeoPlan Center using data derived from multiple sources [51]. This model provides land elevation in meters above sea level in a continuous raster at a 5-meter resolution. In addition, the DEM was used to create a slope raster in ArcGIS Pro which represents the degree of steepness of the terrain in the study area.

### Ecological niche modeling

BRT, RF, and Maxent are machine learning algorithms used frequently for ENM. BRT and RF models both utilize classification and regression trees combined with boosting and bagging principles, respectively, to create an ensemble of trees that improve model performance and fit [52, 53]. BRT and RF models are capable of fitting non-linear relationships and show little impact from data outliers or missing data from predictor variables [52, 53], making them ideal for ecological modeling. BRTs are an additive model, creating trees one at a time, with each new tree fit to residuals present in the previous tree. The algorithm then aggregates the results from each step and a weighted vote is used for prediction [54]. RF uses a different approach than BRT in that it creates trees in parallel, using a random sampling of the data. This results in several trees using bootstrapped inputs with an output selected through the majority vote of the results from each decision tree [53].

Maxent is unique in that it was developed specifically for modeling presence-only species distributions [2]. Maxent develops a model based on the null hypothesis that the target species distribution probability is uniform across the defined study area and moves away from this distribution to the extent required by the constraints imposed by functions of the predictor variables [2]. While it is likely that several distributions will fulfill the imposed constraints, the final model selected is the one representing the distribution with maximum entropy [55].

**Fig 1:**
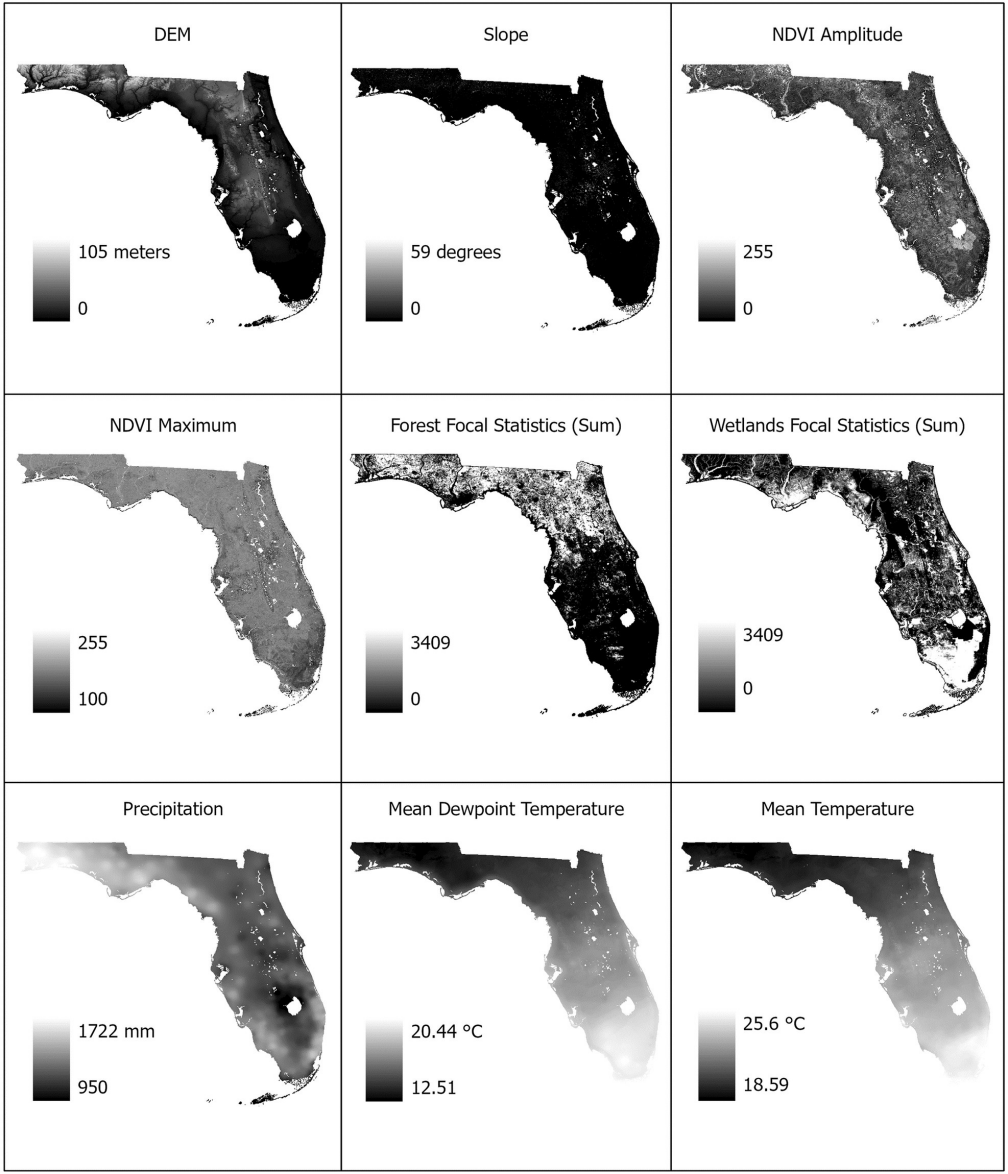
Predictor variable rasters. The above rasters represent the environment spatial variables used for this study. The Digital Elevation Model (DEM) is expressed in meters above sea level. Slope is expressed in degrees from 0 to 90. NDVI amplitude and maximum are unitless and based on NDVI units. Forest and wetland focal statistics indicate the sum of the cells within a 1000-meter circular neighborhood with forest or wetland characteristics, respectively. Precipitation is expressed in millimeters. Mean dewpoint temperature and mean temperature are expressed in degrees Celsius.

Rasters representing predictor variables must match with regard to their coordinate system, extent, and cell size for use in ENMs. To this end, each raster was masked to the study area and extent defined by the DEM raster, projected to the 30-meter cell size of the NLCD raster, and transformed to the USA Contiguous Albers Equal Area Conic projected coordinate system using the Extract by Mask function in ArcGIS Pro. The 30-meter cell size was selected as it represents the native resolution of the NLCD raster, provided sufficient climate and environmental variability for analysis at the county level, and it afforded an area easily relatable to the flight range of the vectors. Cell size re-projection occurred through interpolation using nearest neighbor resampling. The USA Contiguous Albers Equal Area Conic projection was selected as it is well suited for mapping of locations extending east to west in mid-latitude regions, provided an equal area map of the study region, and used meters as a unit of measurement in ArcGIS Pro [56]. Fig 1 presents each of the rasters utilized in this study.

The 269 presence points were examined and found to exhibit strong spatial autocorrelation (SAC) using the Global Moran’s I geoprocessing tool in ArcGIS Pro. To reduce effects of SAC and selection bias on our models spatial thinning, a process in which a subset of locations is randomly selected in geographic space, was applied to the presence locations. Spatial thinning has been shown to decrease model overfitting and improve performance in studies where the presence records exhibited selection bias [57, 58]. Spatial thinning in geographic space generally involves one of two methods. The first involves the use of an equal area grid overlay with a random sampling from within each grid [59]. The second involves the removal of presence records based on a minimum neighbor distance between the remaining records [60]. For this study, the latter method of removal of presence records based on a minimum neighbor distance was selected as it provided a method for controlling selection bias while also reducing SAC. Thinning was conducted in ArcGIS Pro using the Spatially Rarefy Occurrence Data for SDMs tool available in SDMtoolbox. The minimum neighbor distance initial value of 10-km increased by 1-km in each succeeding run. A 15-kilometer minimum neighbor distance was required to reduce SAC to approximately zero while retaining as many presence locations as possible, resulting in 101 presence locations available for model creation.

**Fig 2:**
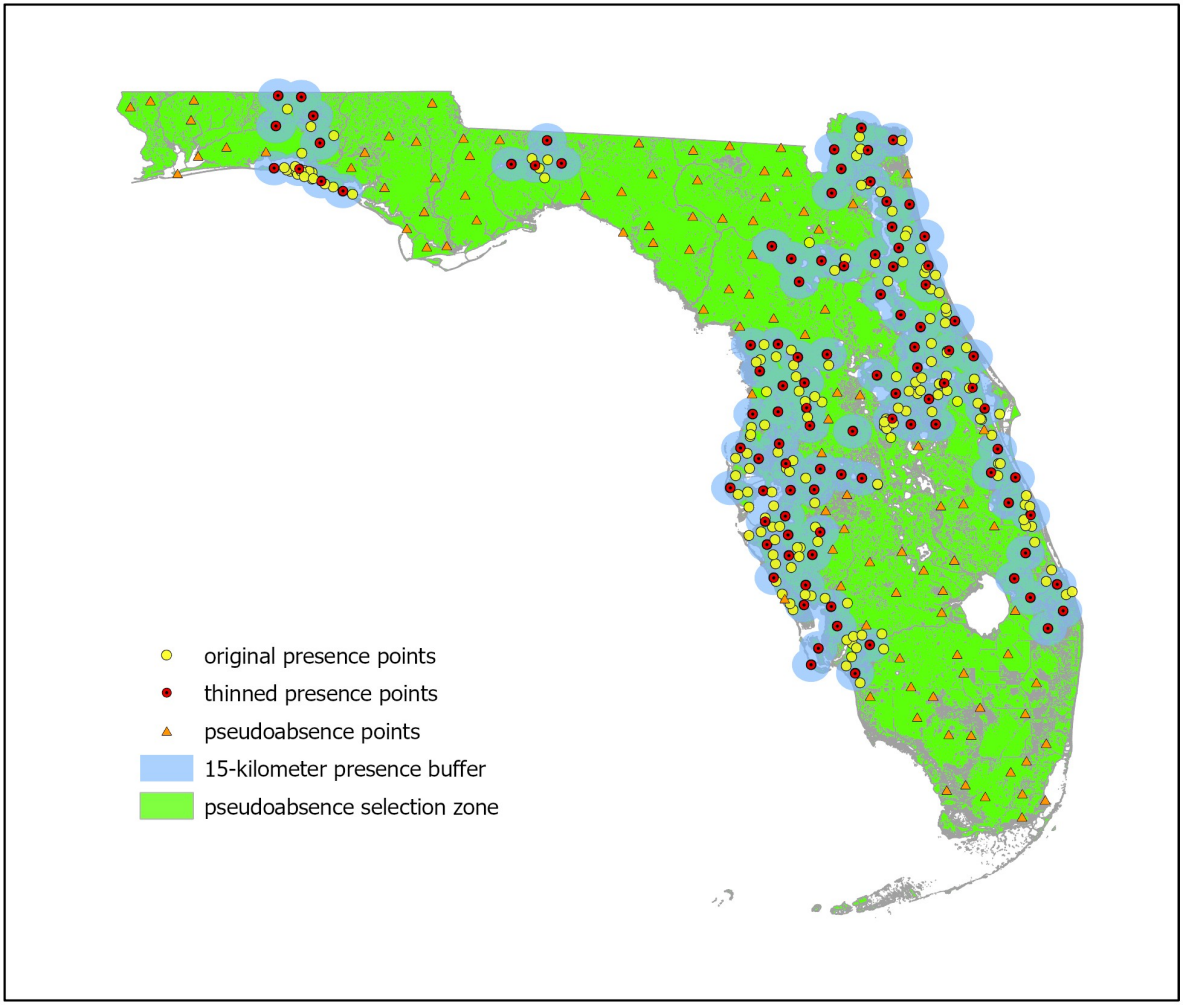
Presence and pseudoabsence points. The original and thinned presence points are indicated within the 15-kilometer buffer used for reduction of spatial autocorrelation. The remaining regions outside of the buffer constitute the pseudoabsence selection zone with the randomly selected pseudoabsence points indicated.

One set of 101 PA points were developed for use in BRT and RF model creation. These points were developed by using the 15-kilometer buffer created during the SAC analysis to mask geographic regions of the study area from PA selection. The create random points tool was used to create a shapefile from the remaining study area while maintaining a minimum of a 15-kilometer distance between PA points to generate 101 PA points. A second set of 10,000 background points was developed for use in Maxent. The create random points tool was used to create a shapefile consisting of 10,000 points selected from the entire study area for background sampling. Both PA and background shapefiles were exported as CSV files for use in R. All rasters were exported in Tagged Image File Format (TIF) for use in R. Fig 2 indicates the original and thinned presence points, 15-kilometer presence buffer, PA selection zone, and PA points.

Raster files were stacked in R to create a RasterStack variable for analysis. Sample with Data (SWD) files were created using presence, PA, and background points along with their respective values extracted from each of the rasters. The SWD files consisted of presence and PA data for BRT and RF use, and presence and background data for Maxent use. Each SWD file was then randomly divided into three separate datasets, a training set with 60% of the presence points, a testing set with 20% of the presence points, and a validation set with the remaining 20% of presence points. The training file was then divided into 4 random folds to allow for k-fold cross-validation training of the models. Initial BRT, RF, and Maxent models were developed with default settings (one exception, Maxent iteration was set for 5000 in ALL models) and using all available predictor variables in R. The withheld testing set was used during model optimization—correlated variable analysis, data-driven variable reduction, and hyperparameter optimization. The withheld validation set was used for validation testing of the final models.

Each model was first tested for correlated variables. Correlated variable analysis identified and removed the predictor variable within a correlated pair as indicated by a Spearman’s correlation coefficient greater than 0.75 that resulted in a higher area under the Receiver Operating Characteristic curve (AUC) value. Next, data-driven variable reduction was conducted to remove predictor variables performing below the 5% threshold based on the permutation importance of each variable to the model. This provided the most parsimonious model, allowing for greater generalizability with minimal loss to predictive power. Hyperparameter optimization (tuning) was then conducted to select model hyperparameters resulting in the greatest AUC for each model. Hyperparameters for optimization included: BRT—number of trees, learning rate, bag fraction, and interaction depth; RF—number of trees, mtry, and node size; and Maxent—feature selection and regularization multiplier. These values were tested using a gridSearch function evaluating each possible hyperparameter permutation and assessing their effect on testing AUC. Once hyperparameters were identified, the training and testing datasets were combined to create a singular training set to be used for final model training. The final models were tested against the withheld validation dataset to determine final model AUC values. A weighted average based upon these AUC values was then used to create the ensemble model.

### Model validation

The ensemble model was validated using the same validation dataset used to validate the final BRT and RF models. This dataset represented 20% of the spatially thinned 101 presence points randomly selected during creation of the training, testing, and validation datasets. The presence and pseudoabsence points from the validation dataset were treated as binary presence and absence values for the purpose of validation. Using the geocoordinates of these points, the predicted habitat probability values for each location was extracted from the ensemble raster. These values were input into the pROC package in R to calculate the AUC of the ensemble model.

## Results

**Fig 3:**
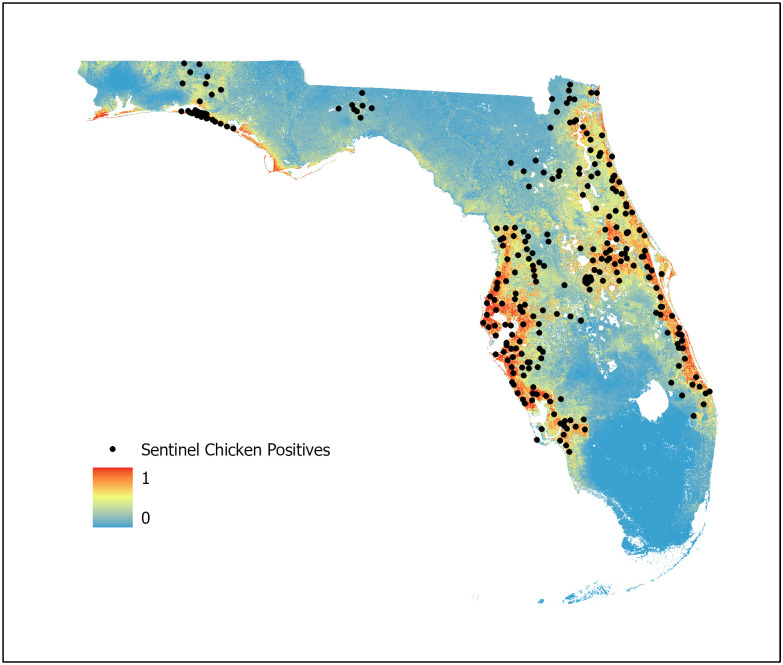
Preliminary model. Preliminary modeling using the complete presence dataset and all available land cover and environmental variables. Model overfitting occurred with selection of developed regions to the exclusion of others.

Pre-study modeling utilizing the complete presence dataset and all available land cover and environmental variables resulted in all models prioritizing land cover variables associated directly with human populations (low, medium, and high-density developed land cover) to the exclusion of others. This resulted in extreme overfitting with models selecting developed areas almost exclusively as high probability habitats for WNV. Fig 3 presents the results of this preliminary modeling. The development bias is clear in that the high probability areas appear as highways, parking lots, and other hardened structures. This is likely due to the selection bias and SAC existing in the presence data, resulting from the directed placement of sentinel chicken surveillance sites in and around population centers. Based on these preliminary findings, the decision was made to control SAC and selection bias through spatial thinning of presence records and to select land cover variables specific to the WNV vectors while excluding those associated directly with human development.

The 269 presence locations from the initial dataset exhibited significant SAC (Moran’s I 0.420390, Z-score 10.559132). Geographical thinning to a 15-kilometer spacing reduced SAC to near zero (Moran’s I 0.096243, Z-score 1.291562) while decreasing presence locations to 101.

**Fig 4:**
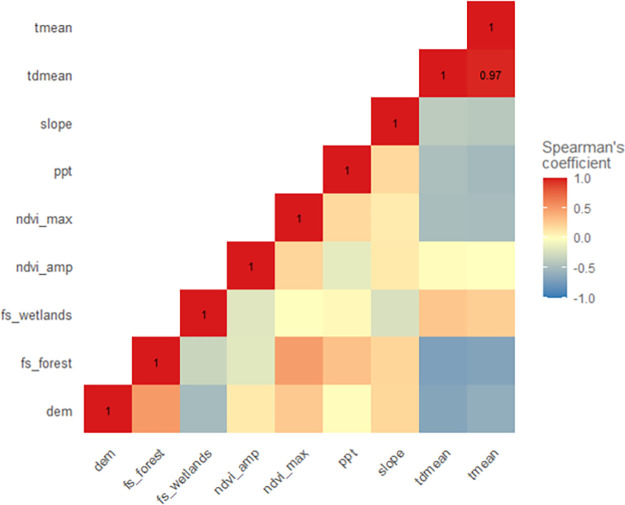
Boosted regression tree and random forest variable correlation matrix. tmean—mean temperature, tdmean—mean dewpoint temperature, slope—degree of slope, ppt—precipitation, ndvi_max—NDVI maximum, ndvi_amp—NDVI amplitude, fs_wetlands—wetlands focal statistics, fs_forest—forest focal statistics, dem—digital elevation model.

**Fig 5:**
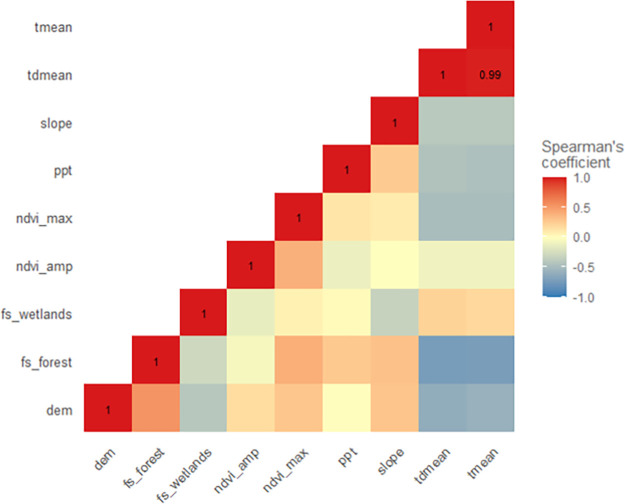
Maximum entropy variable correlation matrix. tmean—mean temperature, tdmean—mean dewpoint temperature, slope—degree of slope, ppt—precipitation, ndvi_max—NDVI maximum, ndvi_amp—NDVI amplitude, fs_wetlands—wetlands focal statistics, fs_forest—forest focal statistics, dem—digital elevation model.

Variable correlation was examined using the PA and background points for each model as applicable. For the BRT and RF models, mean temperature and mean dewpoint temperature exhibited a strong positive correlation (Spearman’s coefficient of 0.97; Fig 4). Mean temperature was selected for removal from the BRT model while mean dewpoint temperature was selected for removal from the RF model. For the Maxent model, mean temperature and mean dewpoint temperature were again found to be highly correlated (Spearman’s coefficient of 0.99; Fig 5). Mean temperature was selected for removal from the Maxent model. All remaining variables were below the 0.75 correlation cutoff selected for this study.

Variable reduction resulted in the removal of no additional variables from the BRT or RF model. Variables ranged in permutation importance from 5.950 (SD 2.517) for NDVI maximum to 31.875 (SD 10.966) for mean dewpoint temperature in the BRT model (Table 1) and from 0.850 (SD 0.661) for DEM to 44.200 (SD 18.191) for mean temperature in the RF model (Table 2). Maxent variable reduction resulted in the removal of NDVI maximum and precipitation. The remaining variables ranged in permutation importance from 6.950 (SD 1.586) for DEM to 35.000 (SD 3.707) for wetlands focal statistics (Table 3).

**Table 1:** Boosted regression tree variable permutation importance.

BRT Variables	Permutation Importance	SD
Mean Dewpoint Temperature	31.875	10.966
NDVI Amplitude	17.700	2.968
Slope	13.875	4.941
Precipitation	11.475	7.961
DEM	6.700	3.700
Wetlands Focal Statistics	6.350	1.674
Forest Focal Statistics	6.000	0.735
NDVI Maximum	5.950	2.517

**Table 2:** Random forest variable permutation importance.

RF Variables	Permutation Importance	SD
Mean Temperature	44.200	18.191
NDVI Amplitude	24.850	14.515
Precipitation	11.900	13.237
NDVI Maximum	8.175	4.102
Slope	6.050	4.622
Forest Focal Statistics	2.975	1.680
Wetlands Focal Statistics	1.050	0.465
DEM	0.850	0.661

**Table 3:** Maximum entropy variable permutation importance.

Maxent Variables	Permutation Importance	SD
Wetlands Focal Statistics	35.000	3.707
Mean Dewpoint Temperature	19.425	6.629
NDVI Amplitude	17.025	0.918
Forest Focal Statistics	14.575	6.824
Slope	7.025	4.456
DEM	6.950	1.586

**Fig 6:**
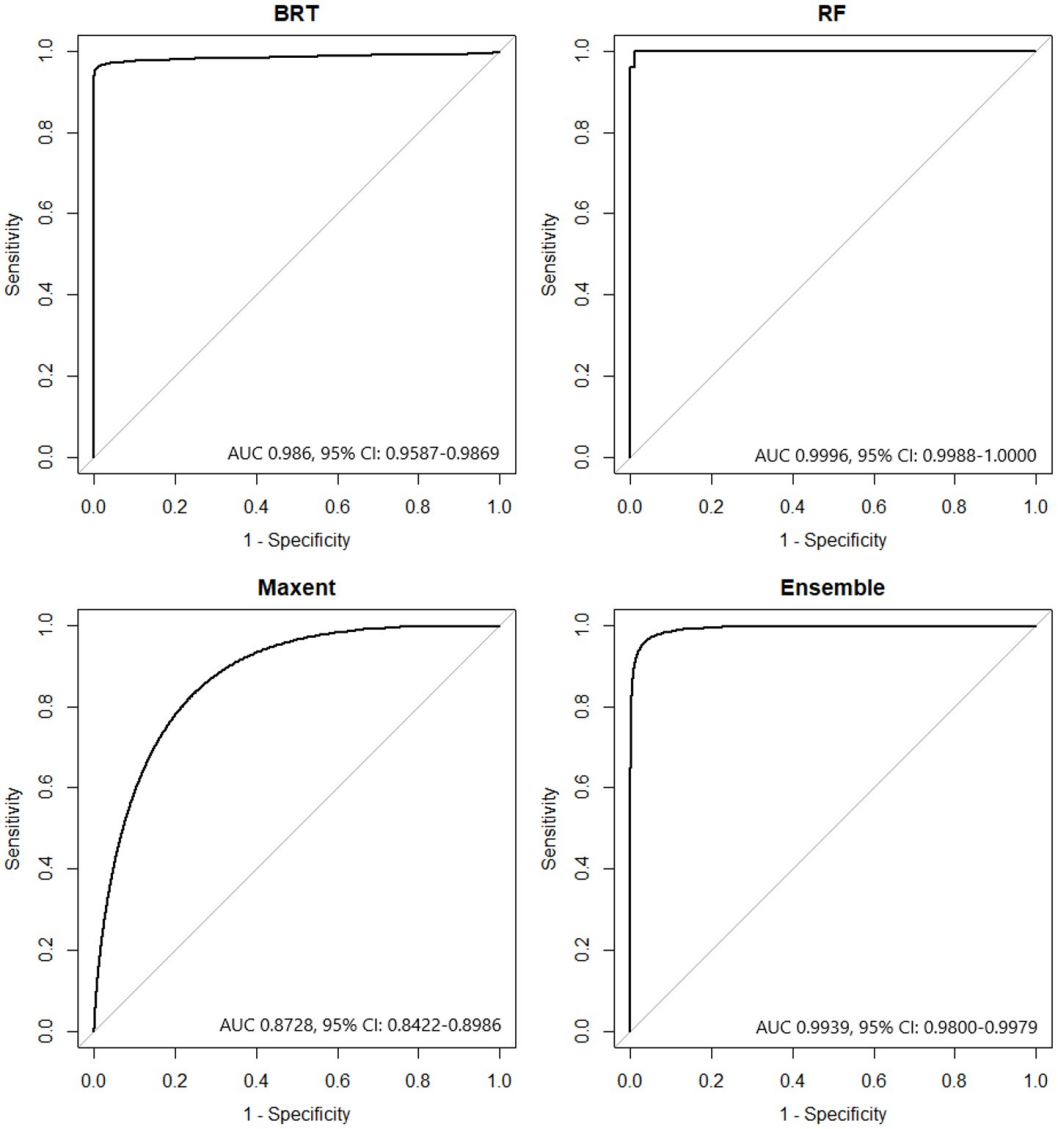
Model receiver operating characteristic curves. BRT—boosted regression tree, RF—random forest, Maxent—maximum entropy. The area under the curve value for each model with its 95% confidence interval is indicated.

AUC was selected as the measure of model predictive power for this study due to its general acceptance as a measure for ENM models as it takes into consideration sensitivity and specificity [61], is considered independent from prevalence [62], and is a threshold-independent measure of model performance [63]. Assessment of AUC values followed the recommendations of Swets [64]: excellent > 0.90, good 0.80–0.90, fair 0.70–0.80, poor 0.60–0.70, and fail < 0.60. Final models tested against their validation datasets resulted in AUC values of 0.986 (95% CI 0.9587–0.9869) for BRT, 0.9996 (95% CI 0.9988–1.0000) for RF, and 0.8728 (95% CI 0.8422–0.8986) for Maxent. The ensemble model was created using a weighted average based on the AUC value of each individual model resulting in an AUC of 0.9939 (95% CI 0.9800–0.9979). The receiver operating characteristic curve with its associated AUC value for each model is shown in Fig 6.

An equal interval analysis of cell statistics was conducted to observe the percentage of cell value similarity between the three individual models. At the 0–0.2 cell value range (the interval indicating the highest similarity), the three models exhibited 28% cell value similarity with this value increasing to 64% at the 0–0.4 cell value range. This congruence was observed with high probability of WNV activity indicated in each model along the barrier islands, the east and west coasts of peninsular Florida, and in the panhandle region along the gulf coast. Habitat probability generally decreased in each model as distance from the coastal regions increased. However, all three models indicated some areas of high probability within the central peninsular regions of the state.

## Discussion

**Fig 7:**
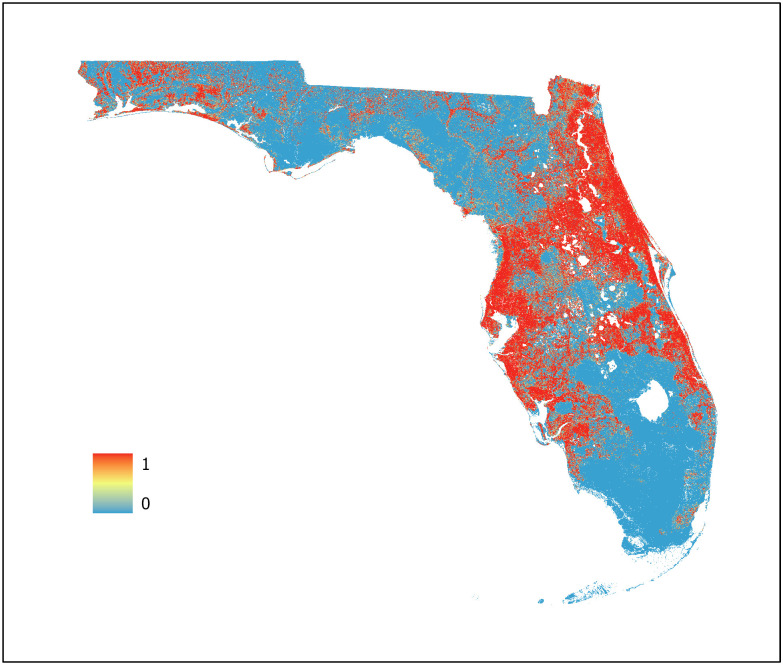
Boosted regression tree model. Predictive values range from 0 to 1 with an AUC of 0.986 (95% CI 0.9587–0.9869).

**Fig 8:**
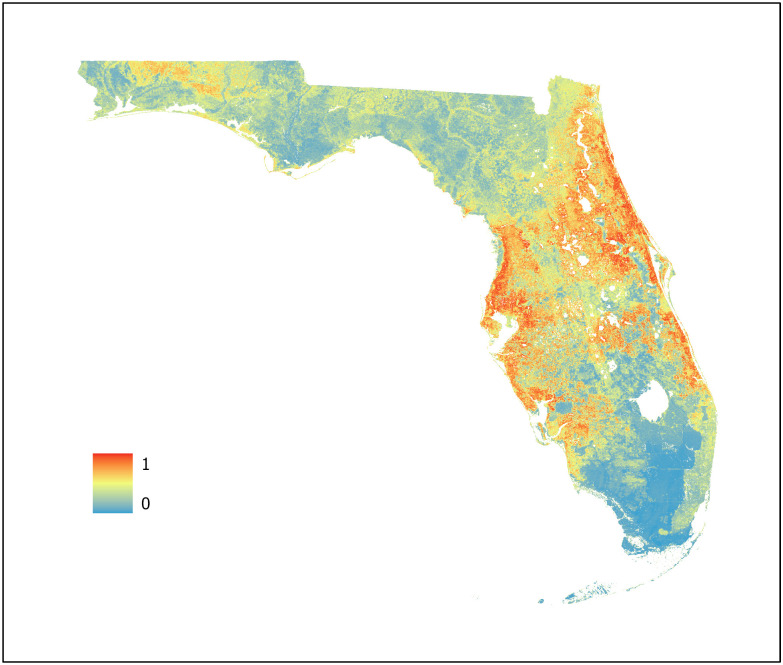
Random forest model. Predictive values range from 0 to 1 with an AUC of 0.9996 (95% CI 0.9988–1.0000).

**Fig 9:**
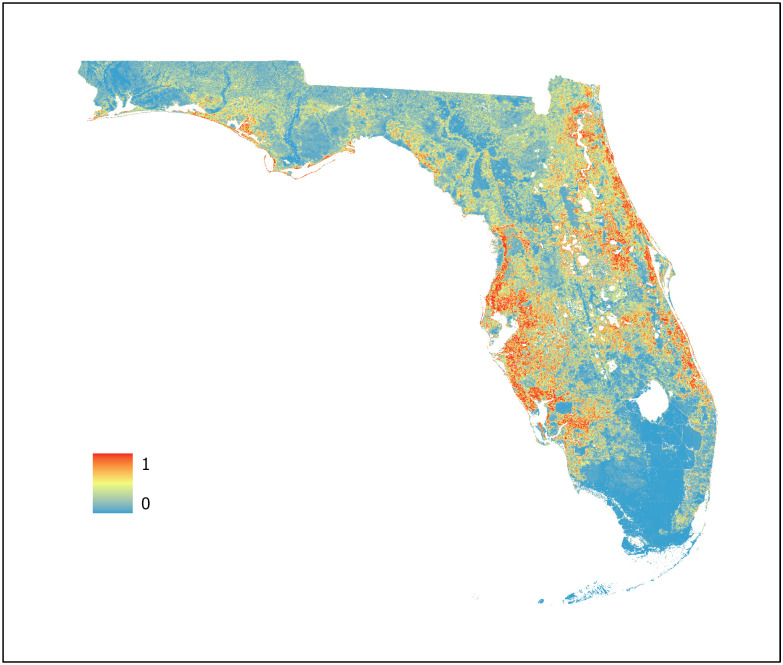
Maximum entropy model. Predictive values range from 0 to 1 with an AUC of 0.8728 (95% CI 0.8422–0.8986).

**Fig 10:**
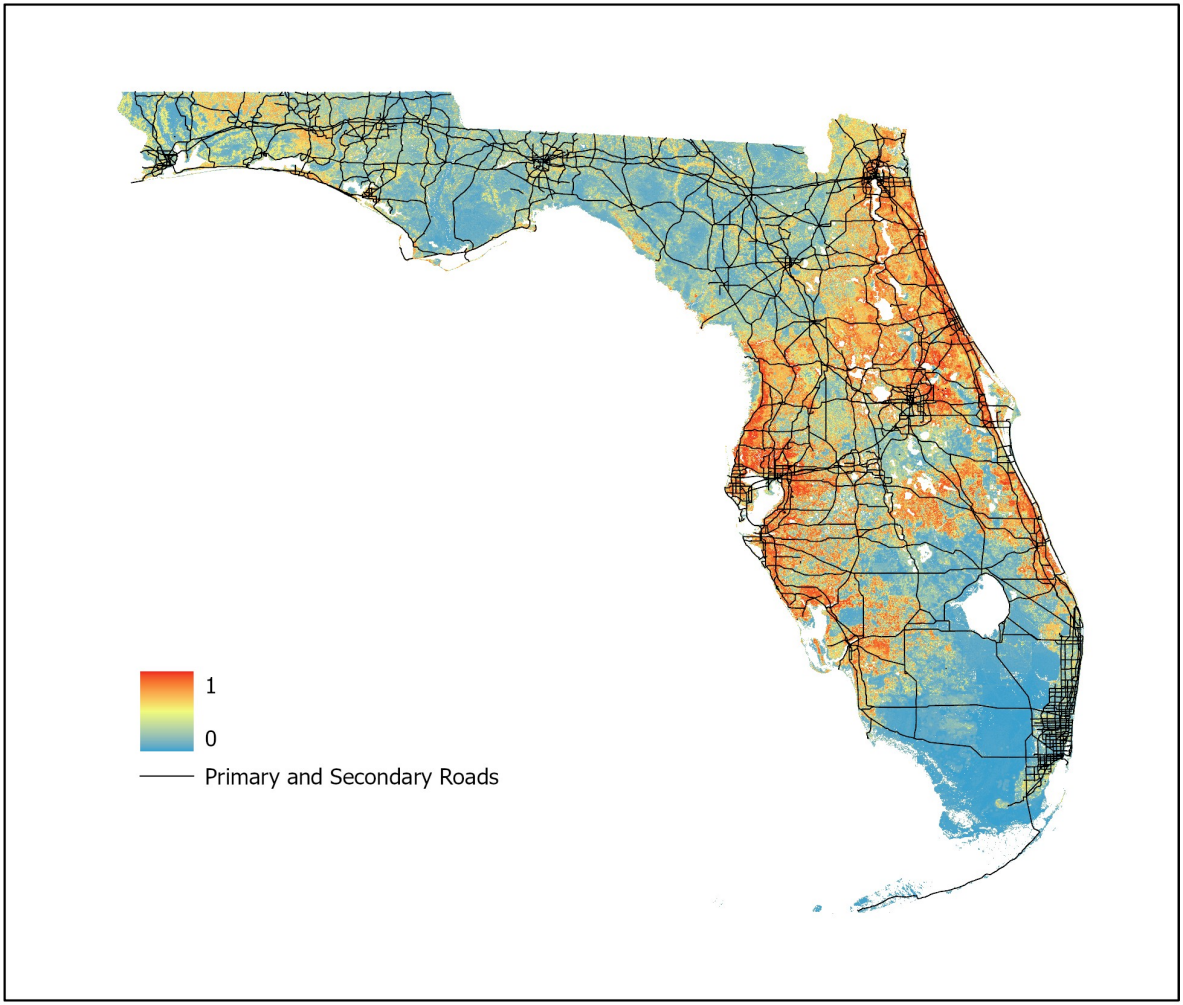
Ensemble West Nile virus model. Predictive values range from 0 to 1 with an AUC of 0.9939 (95% CI 0.9800–0.9979).

For this study, three machine learning models were developed using BRT, RF, and Maxent algorithms (Figs 7–9, respectively) with an ensemble model developed using the AUC weighted average of each individual model to represent WNV habitat probability across the state of Florida (Fig 10). Probability was characterized across the geographic range of the study as a continuous variable from 0 (no probability) to 1 (highest probability). BRT, RF, and Maxent modeling algorithms were selected as they represent the most robust and widely used ENM algorithms currently in use.

The BRT model classified most areas as either very high or extremely low habitat probability, with a limited number of pixels across the study region representing intermediate values. This is possibly due to its iterative process of fitting a new tree to predict the residuals from the previous runs which may result in overfitting of the model. However, overfitting does not necessarily compromise the predictive power of BRT models [54]. This is supported by the equal interval analysis of each map as all three models exhibited similar regions of overlapping high and low probability in conjunction with the high AUC (0.986) of the BRT model. The BRT model used 8 of the 9 predictor variables available. Of these, two were climatic, two NDVI, two geophysical, and two land cover indicating that the BRT algorithm used a variety of predictor variables types during model training to determine WNV habitat probability.

The RF model provided the most gradual change in pixel value between areas of low and high probability across the geographic region. This is readily evident in the panhandle region and slightly less so in the central peninsular region as indicated by the number of intermediate value pixels present. RF models are less subject to overfitting [65] with predictions based on the majority vote of each decision tree. This characteristic of the RF algorithm likely resulted in the smoothing exhibited when compared to the BRT and Maxent models. Like the BRT model, the RF model also used 8 of the 9 predictor variables available with the same variable distribution—two climatic, two NDVI, two geophysical, and two land cover indicating that the RF algorithm also used a variety of predictor variables types during model training to determine WNV habitat probability. However, the permutation importance of the seven common variables varied between the BRT and RF models.

Unlike the extremes exhibited by the BRT model and the smooth transitions between probability values in the RF model, the Maxent model indicated more specific locations for WNV habitat probability values across the geographic space. This is apparent when comparing the panhandle region in each of the three models. The Maxent model provided greater geographic specificity of habitat probability across the range of probability values when compared to the BRT and RF models. Being a presence only algorithm, Maxent is not subject to the error potentially introduced through the use of PA points. This combined with Maxent’s use of thousands of background points to characterize the entire study region likely allows for improved spatial differentiation when compared to BRT and RF. Unlike the BRT and RF models, Maxent used only 6 of the 9 predictor variables available. Of these, two were land cover, one climatic, two geophysical, and one NDVI indicating that the Maxent algorithm also used a variety of predictor variables types during model training to determine WNV probability.

The ensemble model, developed using the weighted mean of the AUC values of the BRT, RF, and Maxent models provides a model that leverages the advantages of each individual machine learning algorithm while reducing the uncertainty present in an individual model. Studies indicate that ensemble modeling methods can provide significant improvement in model accuracy over individual models [66, 67]. The ensemble model created for this study exhibited many of the desired traits from individual models—definitive identification of regions exhibiting high probability of WNV activity, high geographic specificity of WNV habitat across the probability spectrum and smoothing between areas of high and low probability—while minimizing undesired traits such as potential overfitting and the limited number of intermediate probability value pixels. This supports the study concept that no individual algorithm is likely to provide an ideal model and that an ensemble modeling technique is useful to improve predictive value and generalizability. As each model contributing to the ensemble minimized the number of predictor variables necessary for training, the resulting ensemble model represents a parsimonious model allowing for improved generalization. To further improve the operability of the ensemble model, it was fitted with a shapefile indicating the primary and secondary roads of Florida allowing MCPs to select sentinel chicken surveillance sites in high-probability locations that are reasonably accessible to allow for sentinel chicken testing and coop maintenance.

Florida is divided into three Level III Ecoregions as defined by the United States Environmental Protection Agency based on the framework of James Omernik [68, 69]. One of these ecoregions, the Southern Florida Coastal Plain, encompasses the southern tip of Florida. It is largely occupied by the Everglades National Park, Big Cypress National Preserve, and the city of Miami. This region is ecologically unique and is dominated by ecological systems (e.g., herbaceous wetlands) that are rare in the rest of the state. In addition, there is no sentinel chicken sampling within this ecoregion. Our model predicts that this region, including the Miami-Dade metropolitan area is a low to moderate risk area for WNV. This is likely due to the high level of urban development in this area, which though densely populated, is not ideal habitat for WNV’s vectors. However, our model results in this ecoregion should be considered with caution as the model is extrapolating beyond the bounds of the data used to develop it.

Given the geographic range of the study, the absence of statewide sentinel surveillance and the county or municipal-level operational foci of MCPs, the resulting distribution of surveillance locations across the state exhibited a high level of SAC. Of the MCPs conducting sentinel chicken surveillance, the varied degree of scientific rigor involved in the selection of sentinel surveillance sites and desire for ease of sampling and maintenance at the surveillance location introduced potential selection bias [70]. The potential impact of SAC and selection bias on model development is further compounded through the use of presence only data [71–73].

Many ENMs utilize both presence and absence records in model creation. BRT and RF are both examples of this type of model. Ideally, we would have used both confirmed presence and confirmed absence data in the developments of our model. However, the model was developed using data from sentinel chicken data and the number of chicken coops found to be in an area where WNV was absent (as defined by no positive chickens in the five-year period used to develop the model) were very limited. In fact, in a comprehensive analysis of coop locations, we were only able to identify a single coop that had no WNV activity in the five-year period of this study. This is probably due to the fact that coop locations were originally sited near confirmed cases of SLE. SLE, like WNV, is a flavivirus and WNV and SLE share many characteristics including using the same mosquito species as vectors. Thus, it is not surprising that almost none of the coops were located in areas where WNV was absent. Since true absence data were not available, we relied on PA data to develop the models.

PA points are commonly used in ENM, demonstrated in the literature both by papers related to the development of PA methodology [74–87] and their use in studies for which absence data is unavailable [75, 77, 78, 80, 81, 86, 88–92]. Given the potential impact of false negative data on model development, great consideration was given during the development of the PA points. To reduce this potential impact, both the location and number of PA points must be carefully considered [93, 94]. In this study, a geographic space for PA selection was delineated as a minimum distance of 15-km from the thinned presence points used in the study with a minimum distance of 15-km between PA points. This space maximized the separation between presence and PA locations while allowing for the selection of 101 PA points to equal the number of thinned presence points used in the study. This methodology is further supported by the fact that the disease vectors are a common species within Florida. As such, the effects of few potential false-negatives should be offset by the presence data [75, 81]. In addition, Maxent as a presence-only model makes use of thousands of randomly selected background points to characterize the study region rather than the PA points developed for use with the RF and BRT models. The regions of high and low productivity characterized by the Maxent model share a high degree of congruence with the RF and BRT models as indicated by the equal area analysis which supports the overall accuracy of the models and in turn the use of PA points. However, the use of PA points is recognized as a limitation in the study methodology.

Despite being a presence-only method by design [95], Maxent still requires the use of background data from the study region for model development. Maxent by default selects 10,000 background locations from the study region to characterize the environmental background of the study area [95]. Recommendations for guided selection of background points for use in Maxent exist, but for the purpose of controlling bias present in the sampling records [72, 96]. As the potential selection bias in this study was controlled with spatially thinned presence records, the default value of 10,000 random background points was used.

The selection bias present in the complete sentinel chicken dataset was readily evident due to the high spatial autocorrelation of the coop locations. Initial sentinel chicken surveillance locations were selected near documented human cases of SLE. Subsequent surveillance locations were selected in and around human population centers to determine potential arboviral threats to public health, but were placed near roads to simplify sampling and maintenance. This resulted in the majority of coop placements occurring within developed land cover regions to the exclusion of other land cover types known to be WNV vector habitats. During preliminary modeling, this was readily evident with clearly overfitted models selecting roadways and other hard surface locations to the exclusion of other land cover types. To control for this selection bias, the sentinel chicken surveillance data was spatially thinned until the spatial autocorrelation of the data reached near zero. Furthermore, the categorical NLCD raster was processed into separate continuous variable rasters for each land cover type. Land cover variables associated with WNV vector habitat were selected for use while excluding those associated with human habitation.

Another potential limitation in this study was the use of the BRT and RF validation dataset to determine the AUC of the ensemble model. This data was previously used to determine the AUC of the BRT and RF models. This AUC value was used as the weight for the weighted average used to create the ensemble model. This introduces a potential bias in the determination of the ensemble AUC. However, use of an unused sentinel chicken dataset representing a different year or period for AUC computation could potentially introduce its own set of biases. As such, it was determined that this method provided an acceptable and low probability of AUC error. The Maxent validation dataset was not considered for ensemble validation as Maxent is a presence-only model that uses thousands of background points to characterize a study region. It therefore was not appropriate for use as a binary presence/absence dataset for AUC calculation and model validation.

Despite the limitations discussed above, the use of sentinel chicken data has advantages that are derived from both its numbers and its location of infection accuracy. During the period of this study, 2012 sentinel chickens tested seropositive for WNV at 269 locations across Florida. By comparison, Florida Health Department Records for the same timeframe indicate only 31 equine cases and 76 human cases acquired in Florida. Furthermore, sentinel chicken coop locations are precise (verified by GPS) and stationary. When a sentinel chicken tests seropositive for WNV, the coop is the known site of exposure and infection. Equine and human WNV data is not as definitive, as the site of WNV exposure is generally difficult to determine. Location data is generally a coordinate representing a centroid in a horse pasture or owners’ residence for equine data [97, 98] and a county or zip code for human data [99, 100]. These locations are at best an approximation due to animal/human movement resulting in an indeterminate location of exposure.

Future studies would ideally involve the movement of existing chicken coops or placement of new chicken coops into areas of high and low probability to field validate the model. A possible alternative due to the logistical requirements of chicken coops would be mosquito collection and pool sampling for WNV in the same high and low probability areas.

This model addresses limitations present in many existing ENMs while reducing error and improving predictive power through creation of an ensemble model consisting of three individually trained machine learning algorithms. A similar ensemble methodology could be applied to existing arboviral models to improve overall accuracy which may also allow for the development of a multi-virus arboviral habitat probability model. This ensemble model will allow MCPs to optimize placement of sentinel chicken coops for probability-based surveillance of WNV, making better use of finite resources and potentially reducing operating costs. More importantly, the model will improve WNV surveillance allowing for earlier detection of virus transmission facilitating a more rapid, targeted vector control response in turn reducing the potential for disease transmission to human or animal.
